# PLK1 Inhibition Induces Immunogenic Cell Death and Enhances Immunity against NSCLC

**DOI:** 10.7150/ijms.60135

**Published:** 2021-08-19

**Authors:** Jie Zhou, Qifan Yang, Lisen Lu, Zhan Tuo, Zhexing Shou, Jing Cheng

**Affiliations:** 1 Cancer Center, Union Hospital, Tongji Medical College, Huazhong University of Science and Technology, Wuhan 430022, P. R. China.; 2 Department of Integrated Traditional Chinese and Western Medicine, Union Hospital, Tongji Medical College, Huazhong University of Science and Technology, Wuhan 430022, P. R. China.

**Keywords:** PLK1, ICD, immune infiltrates, NSCLC

## Abstract

PLK1 inhibitors were shown, *in vitro* and *in vivo,* to possess inhibitory activities against non-small cell lung cancer (NSCLC), and such inhibition has been proven by clinical trials. However, it remains unclear whether and how the immune microenvironment is associated with the action. In this study, we found that inhibiting PLK1 could alter the tumor immune microenvironment by increasing DC maturation, and enriching T cells infiltration. PLK1 inhibitors, serving as immunogenic cell death (ICD) inducers, indirectly activated DCs, instead of directly acting on DC cells, through the surface expression of costimulatory molecules on and enhanced phagocytosis by DCs. Furthermore, upon targeting PLK1, tumor cells that had undergone ICD were converted into an endogenous vaccine, which triggered the immune memory responses and protected the mice from tumor challenge. Collectively, these results suggested that the PLK1 inhibitor might function as an immune modulator in antitumor treatment.

## Introduction

PLK1, a member of polo-like kinase (PLK) family, serves to control the checkpoint of cell division, a process indispensable in complete mitosis 1. PLK1 has been found to be over-expressed in multiple cancers, including NSCLC, and the upregulated PLK1 is indicative of an unfavorable prognosis 2-4. Furthermore, some studies showed that PLK1 expression level could be a reliable measure of risk for metastasis 5, 6. These findings suggested that the PLK1 is implicated in carcinogenesis, and might potentially be used as a therapeutic target.

As members of serine/threonine kinase family, which target kinesins and kinases in mitosis, PLK1 and aurora kinase A (AURKA) share some cellular phenotypes and as a result, the effects of PLK1 inhibition and AURKA blockade might overlap 7. Recently, inhibiting AURKA was shown to modulate the immune microenvironment by regulating immunosuppressive myeloid cells 8. However, whether PLK1 blockade can initiate immunity remains unknown.

BI2536, a selective PLK1 inhibitor, was shown to have a wide array of cytotoxic effects on various tumors upon binding to the kinase domain of PLK1 and arresting the mitosis cycle 9. In general, BI2536 promotes apoptosis and mitotic cell death, and the dead cells were cleared primarily by macrophages, which leads to a non-inflammatory event 10, 11. Recently, Wu *et al*. confirmed that BI2536 could induce pyroptosis, a form of programmed necrosis, and worked as a powerful trigger of antitumor immunity 12, 13. Furthermore, an analysis of high-throughput data regarding pan-cancer immunity from TCGA revealed that the anti-tumor efficiency of BI2536 was positively correlated with the infiltration of immune cells, which suggests that it might be an immunity regulator 14.

Immune response has been demonstrated to be an important factor in tumor progression and treatment. Effectively activating antigen-specific immune response requires targeting the initial stages of “cancer-immune cycle”, including release, capture and presentation of antigens 15. Several cytotoxic agents have been tried to modulate antigen phagocytosis and presentation of DCs, such as oxaliplatin, anthracyclines and ionizing radiation, which are able to render the tumor antigens “visible” to immune system, *i.e*., inducing ICD of cancer cells, and, consequently, a robust adaptive immune response 16-19. ICD is characterized by the endoplasmic reticulum stress response (translocation of calreticulin (CRT) to surface expression), which sends an “eat me” signal and activates phagocytosis of tumor cells by DCs 20. In addition, ICD-associated release of ATP and high-mobility-group box1 were found to attract and activate DCs, making ICD a promising candidate for initiating antitumor immune response 21. On the basis of these findings, two questions present themselves: Will PLK1 blockade help trigger the antitumor immunity? Is ICD the major contributor to the initiation?

In this study, we demonstrated that PLK1 blockade indirectly, rather than directly, elicited DC maturation, which triggered a robust antitumor T cell immune response in an established lung transplantation tumor. Further studies revealed that PLK1 inhibitors served as an ICD inducer to expose the markers of ICD, which enhanced the DC phagocytosis and activated DCs, and helped protect mice from tumor challenge. Collectively, our findings suggested that PLK1 inhibitors might function as immunomodulators, which make them more attractive as anti-NSCLC agents.

## Methods

### Data Collection and Bioinformatic Analysis

The TCGA data regarding transcriptome expression profiles of 50 cases of healthy subjects and 495 cases of lung adenocarcinoma (LUAD) were download from public databases (https://tcga-data.nci.nih.gov/tcga/tcgaDownload.jsp). Kaplan-Meier survival analysis of OS in LUAD was performed in terms of PLK1 expression. DAVID 6.8 and KEGG were used for Gene annotation and pathway analysis 22. Gene Set Enrichment Analysis (GSEA, https://www.broadinstitute.org/gsea/) was conducted to investigate the biological functions of PLK1. To verify the effect of PLK1 on immunity, the relationship between PLK1 and immune-cell populations was evaluated by using Microenvironment Cell Populations-counter (MCP-counter) 23. The statistical analyses and picture drawing in this part were performed by using R-3.4.3 and GraphPad Prism 7 software packages.

### Cells and Reagents

Lewis (LLC) murine lung carcinoma cells were purchased from American Tissue Culture Collection (ATCC), and maintained in DMEM medium (Gibico) supplemented with 10% fetal bovine Serum (FBS; Gibico), 10 U/ml penicillin and 10 U/ml streptomycin under 5% CO_2_ at 37 °C.

BI2536, as a PLK1 inhibitor, was procured from Selleck (America). For *in vitro* determination, BI2536 was dissolved in DMSO to make 1 mmol/L stock solution, which was stored at -20 °C and diluted with DMEM culture solution before use. For *in vivo* examination, BI2536 was dissolved in vehicle (in the order of 5% DMSO, 40% PEG 300, 5% Tween 80 and 50% ddH_2_O) and the final concentration was 6 mg/ml.

### Drug-viability Study

LLC tumor cells were detected to estimate their susceptibility to BI2536. LLC cells were seeded into 96-well plates (7000 cells per well). 24 h after seeding, cells were incubated with increasing concentrations of BI2536 for 24 h. Cell proliferation was measured by using the CCK-8 assay kit (Biosharp).

### CRT assays

Tumor cells (5×10^5^ cells per well) were planted into a 24-well plate precoated with gel (PureCol® EZ Gel, Sigma, 1:150) and treated with BI2536 for 24 h. To analyze the translocation of CRT in immunofluorescence, the samples were fixed in 4% paraformaldehyde (PFA) before permeabilization with 0.1% TritonX-100, and then stained with a rabbit anti-mouse anti-CRT antibody (ab92516, Abcam, 1:100) for 30 min. Subsequently, the cells were stained with a secondary antibody FITC goat anti-rabbit IgG (GB22303, Servicebio, 1:200). The cytoskeleton was incubated with phalloidin (C2207S, Beyotime, 1:1000) and cell nuclei were stained with 4′, 6-diamidino-2-phenylindole (DAPI, Beyotime). The fluorescence information was acquired by laser confocal microscopy (NIKON Eclipse Ti). For flow cytometry, treated tumor cells were incubated with anti-CRT and secondary FITC goat anti-rabbit IgG as previously described. Then the samples were prepared into 100 μL of PBS suspension and subjected to FACScalibur detection.

### ATP Release Detection

LLC cells were seeded into 6-well plates and after having grown to 70-80% confluence, cells were incubated with serial dilutions of BI2536 for 24 h. The cell culture supernatants were collected for quantification of the extracellular ATP by employing an Enhanced ATP Assay Kit (Beyotime).

### Mouse Bone Marrow-derived DCs Activation and Phagocytosis Assays

Mouse bone marrow-derived DCs (BMDCs) were extracted from the femurs of male C57BRL/6 mice (eight to twelve weeks old) and cultured in RPMI 1640 complete culture medium containing GM-CSF (20 ng/ml, Biolegend) for 7 days. The suspended BMDCs were half-changed on the 3^rd^ and 5^th^ day, respectively. To determine the phagocytosic ability and activation status of BMDCs after direct treatment with BI2536, BMDCs were incubated with a series of concentrations of BI2536 for 24 h. Then, LLC cells were stained with CFSE (Biolegend, 5 mM, 1:1000) and co-cultured with BMDCs at a ratio of 1:1 in the normal culture medium for another 24 h. To determine the phagocytosis status of BI2536-treated cancer cells, LLC cells were labeled with CFSE (Biolegend, 5 μM) and treated with BI2536 for 24 h. Then the treated LLC cells and the induced DCs were co-cultured at a ratio of 1:1 for another 24 h, as indicated in Fig 4A. DCs were labeled by CD11c (117309) fluorescence-labeled Ab, and the fluorescence data of CD80 (104708), CD86 (105012) and MHCII (107630) were used as indicators of the DC maturation. Tumor cell phagocytosis was quantitatively determined by detecting CFSE (tumor)/CD11c (DC) double-positive signals. For immunofluorescence staining, tumor cells were incubated with CFSE for 5 minutes and co-cultured with DiI (C1036, Beyotime, 10 µM)-stained DC cells for 0-2 h at a 1:1 ratio. The cells were fixed in 4% PFA for 20 min and then stained with DAPI, and the fluorescent images were acquired on the Olympus FVMPE-RS platform after a series of washes.

### Mice and Model Animal Experiments

Six-week-old C57BL/6 male mice were procured from CTGU University Laboratory Animal Center (Hubei, China) and were kept in our SPF (specific-pathogen free) animal facility. All animal experiments were performed in accordance with the protocols established in the Animal Experimentation Ethics Committee of the HUST (Huazhong University of Science and Technology, Wuhan, China) and were approved by the Hubei Provincial Animal Care and Use Committee, Hubei, China.

With the LLC subcutaneous xenograft model, 1×10^6^ LLC cells in 50 μl PBS were inoculated subcutaneously into the right flank of C57BL/6. Seven days after the tumor inoculation, mice were randomized into two groups and intraperitoneally injected 100 μl reagent (solvent or BI2536 at 30 mg/Kg) twice a week over a period of two weeks. To evaluate tumor growth, tumor size (*V*) was measured every 3 days by using digital calipers and recorded as volumes (mm^3^) that were calculated according to the formula: *V*=0.5 × length (*L*) × width (*W*)^2^. All the animals were euthanized when the neoplastic lesions exceeded 1000 mm^3^.

In the immunization study, 3×10^6^ LLC cells, either frozen-thawed (unable to activate DCs *in vitro*) 21 three times in liquid nitrogen or treated with 20 μM BI2536 (being frozen-thawed as aforementioned after BI2536 treatment in case of incomplete killing), were inoculated into the left flank of C57BL/6 mice. After 8 d, 4×10^5^ live LLC cells were injected into the contralateral flank, and the tumor incidence and tumor growth were regularly monitored every 2-3 days for the following weeks as indicated in Fig. 3G.

To measure* in vivo* DC activation in lymph nodes, 3×10^6^ LLC cells, either freeze-thawed three times in liquid nitrogen or treated with 20 μM BI2536 (being freeze-thawed as described above after BI2536 treatment in case of incomplete killing), were subcutaneously injected into the tail root of mice at multiple points. 12 h, 24 h, 48 h, 72 h after the treatment, bilateral inguinal lymph nodes were isolated for DC maturation measurement.

### *In vivo* Immunomonitoring

To determine immune cell populations, the detached tumors were weighed, cut into pieces and digested with enzymes, including collagenase V (0.32 mg/mL, Biosharp) and hyaluronidase (0.5 mg/mL, Biosharp) for 1 h. After addition of the RBC lysis buffer for 8 minutes at 4 °C, single-cell suspensions were acquired through 200-mesh filters (the single cell suspension of the lymph nodes was directly harvested through the filter). The cells were incubated with FcR blocking reagent (101320) to block non-specific binding sites, and then stained with anti-mouse fluorochrome-labeled Abs against Zombie Violet™ Fixable Viability Kit (423114), Zombie NIR™ Fixable Viability Kit (423106), CD45 (103130), CD3e (100306), CD4 (100422), CD8a (100751), CD11c (117310), CD86 (105012), CD80 (104708). For analysis of T-cell functions, cells were stimulated with Monensin sodium salt (ab120499, Abcam, 1.5 μg/ml), PMA (ab12029, Abcam, 100 ng/ml), and lonomycin calcium salt (5608212, peproTech, 1 μg/ml) for 5 h. Different from surface staining antibodies mentioned above, intracellular IFN-γ (505808) was detected after fixation and permeabilization. All the flow cytometrical detections in this study were carried out on a flow cytometer (Beckman CytoFLEX S). All antibodies (as listed above) were purchased from Biolegend.

### Statistical Analysis

All statistical analyses were performed by using GraphPad Prism software. For comparison between two groups, unpaired two-tailed Student's *t* test was used, while for comparisons among three or more groups, one-way ANOVA was employed. All the results in this article were presented as mean ± SEM, and *P* values were indicated as * *P* < 0.05, ** *P* < 0.01, *** *P* < 0.001, and NS: not significant.

## Results

### PLK1 expression was correlated with clinical features and immune infiltration levels in LUAD

**Figure 1:**
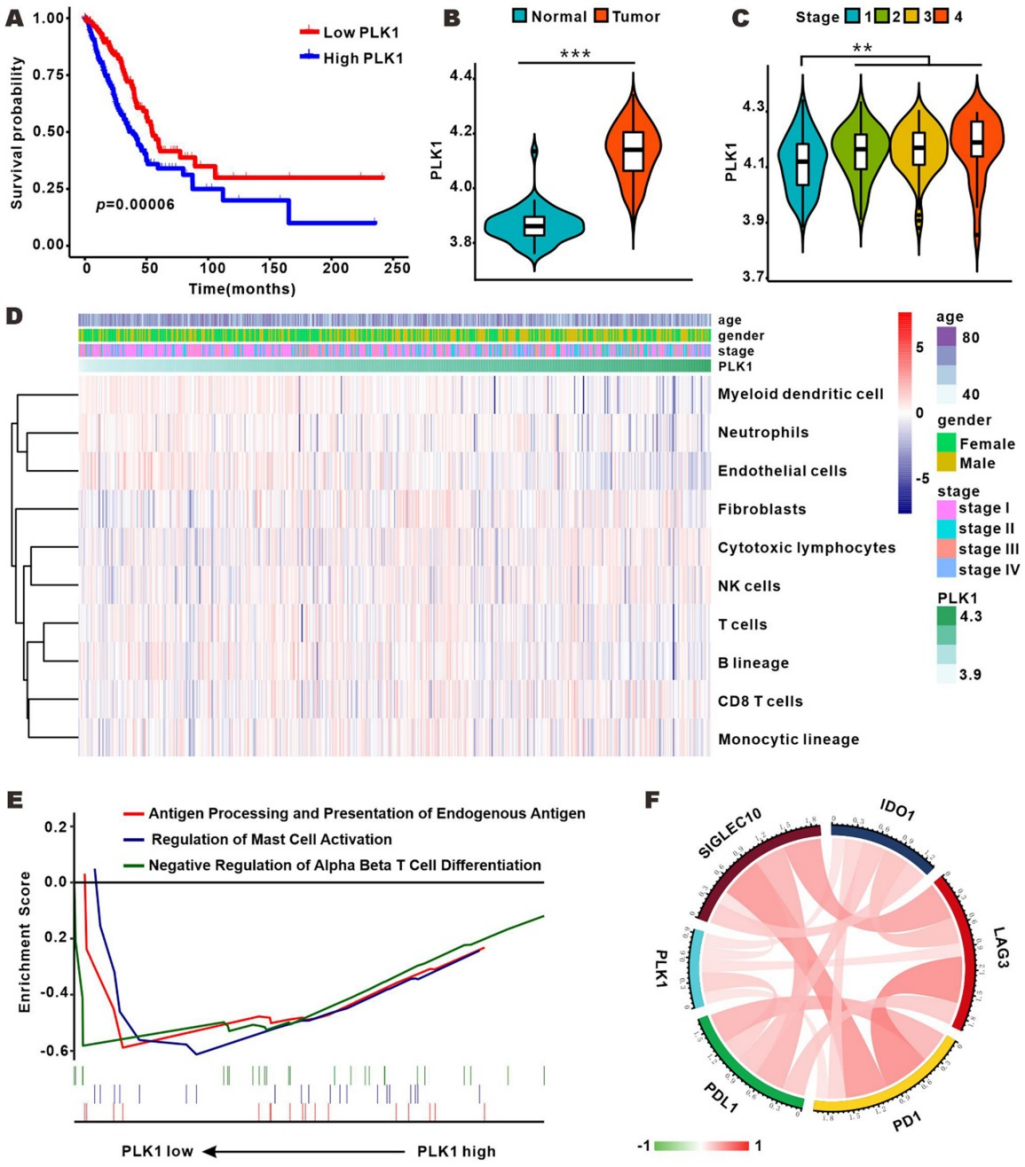
** PLK1 expression was correlated with clinical features and immune infiltration levels on the basis of the TCGA dataset. A.** Kaplan-Meier survival curves showing the relationship between PLK1 mRNA level and overall survival (n=545). **B-C.** Comparison of PLK1 expression between normal and tumor tissue and from stage 1 to stage 4. **D.** Association between PLK1 expression and immune cell populations in LUAD. **E.** Significant enrichment of immunity-related bioprocesses as determined by GSEA analysis. F. The correlation of PLK1 with the expression of several vital immune checkpoints. The information regarding related genes is detailed in Table S5.

To profile the expression of PLK1 in LUAD, we analyzed the RNA-sequence data of 50 normal samples and 495 LUAD samples from TCGA database. As shown in Fig. 1B, PLK1 expression was conspicuously higher in LUAD tumors than in normal samples, and was positively correlated with tumor stage (Fig. 1C and Table S1-4). Furthermore, patients with low PLK1 expression had longer overall survival (OS) than their counterparts with high PLK1 expression (Fig. 1A). These results showed that the higher PLK1 expression was indicative of higher malignancy and more unfavorable prognosis of NSCLC, and were consistent with previous findings obtained with multiple tumors. Cell cycle regulation is generally believed to be the principal mechanism of PLK1-inhibition in antitumor therapy, but whether it impacts tumor immunity *in vivo* is still unknown. MCP-counter showed that there was a weak correlation between PLK1 and immune cells, such as myeloid dendritic cells, T cells and B lineage cells (Fig. 1D and Fig. S1). GSEA was performed to know if the immunity-related biological processes are associated with PLK1 status. The result exhibited that 3 immunity-related biological processes were enriched in the PLK1 low-expression group as compared with high-expression group (Fig. 1E). The three processes involved antigen processing and presentation of endogenous antigen (normalized enrichment score, NES=-1.497, size=17), regulation of mast cell activation (NES=-1.509, size=20) and down-regulation of alpha beta T cell differentiation (NES=-1.307, size=25). Hence, PLK1 in LUAD was negatively correlated with the immune responses. It is widely accepted that immune checkpoints, including multiple costimulatory and inhibitory molecules, play vital roles in the regulation of the activation and effector functions of T lymphocytes 24. We then examined the relationship between PLK1 expression and immune checkpoint-associated molecules (Fig. 1F, Table S5), and found that PLK1 was positively correlated with the critical immune checkpoints, such as PD1, PDL1, LAG3, IDO1 and Siglec10, which release inhibitory signals for T-cell-mediated immunity. These results suggested that the poor prognosis of patients with high PLK1 expression is partially ascribed to the inactivated immune microenvironment, and targeted inhibition of PLK1 might help activate immunity for antitumor therapy.

### PLK1 inhibition triggers antitumor immunity

**Figure 2:**
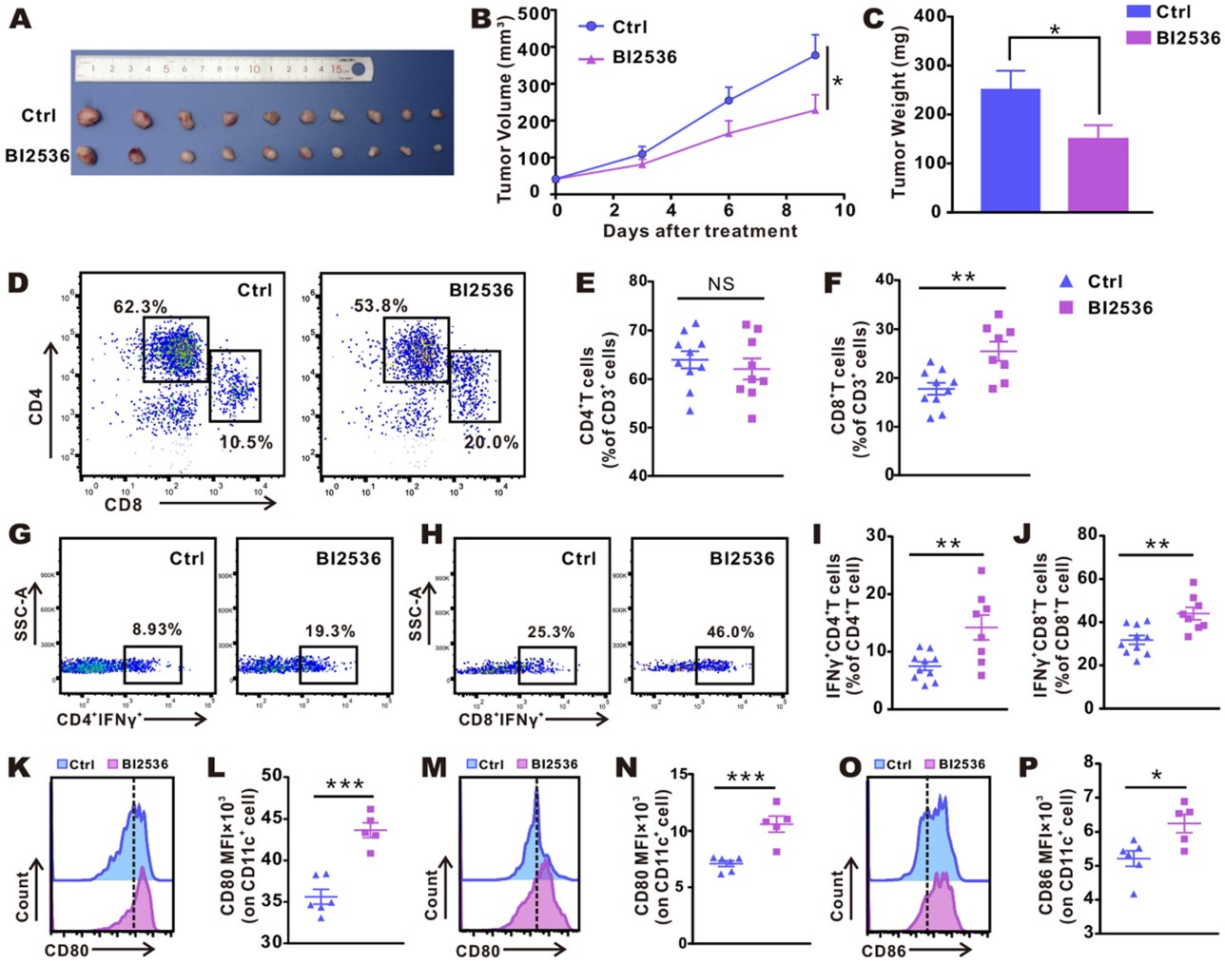
** BI2536 inhibited tumor growth and promoted infiltration and activation of T cells and DC cells. A.** Detached tumor samples after different treatments as indicated. **B.** Tumor growth curves of established NSCLC model treated with repeated intraperitoneal injections of solvent and BI2536 (30 mg/kg). **C.** Quantitative data of tumor weights in each group. Data are presented as mean ± SEM (*n*=10). **D.** Representative plots of flow cytometry. **E-F.** Proportions of CD4^+^T cells and CD8^+^ T cells in the tumor. Data are expressed as mean ± SEM (*n*=8-10). **G-H.** Representative gating strategy. **I-J.** The percentage of Th1 cells and cytotoxic CTLs within tumors in each group *(n*=8-10). **K-P.** Activation status of DCs within inguinal lymph node (K-L) and tumors (M-P) as measured in terms of the mean fluorescence intensity (MFI) of CD80 and CD86 on CD11c^+^ cells. Data are given as mean ± SEM (*n*=5-6).

To validate the antitumor immunity-activating effect of PLK1 inhibition *in vivo*, we used BI2536 to handle an established subcutaneous LLC mouse lung cancer model. As shown in Fig. 2B, with the accumulation of doses, the antitumor effect of BI2536 gradually appeared. When tumors were detached and weighed at the endpoint of the treatment, the tumor load in the BI2536-treated mice was lower (Fig. 2A), achieving a 40% reduction in terms of tumor weight as compared with the control group (Fig. 2C). Of note, no weight loss was observed in any treated mice (data were not shown).

We assessed the status of the immune microenvironment by examining the infiltration and activation of T cells within tumors. Subcutaneous LLC tumor-bearing mice were intraperitoneally administered reagents with or without BI2536 as before, and after the fourth dose, tumors were removed and flow cytometrically was performed (gating strategy is shown in Fig. S2). Fig. 2D-F showed that in BI2536-treated mice, tumor-infiltrating CD8^+^ T cells were significantly increased. Despite a poor response of CD4^+^ T cells to BI2536, the functionally active T cells, including Th1 cells (Fig. 2G-I) and CTLs cells (Fig. 2H-J), were increased significantly, as indicated by elevated percentage of IFN-γ expression. The result suggested that the adaptive antitumor immunity was successfully activated. Moreover, since mounting evidence indicated that DCs play a vital part in T cell-priming, we examined the activation status of DCs within inguinal lymph glands (ILNs, Fig. 2K-L) and tumors (Fig. 2M-P), respectively. As expected, in comparison with matched group, BI2536 treatment resulted in a higher expression of CD80 and CD86, *i.e*., two classical markers of DC activation. Collectively, these findings indicated that BI2536 helps initiate antitumor immunity, principally by enhancing the activation and function of T cells and antigen presenting cells (APCs).

### PLK1 blockade-mediated immunogenic tumor cell death

**Figure 3:**
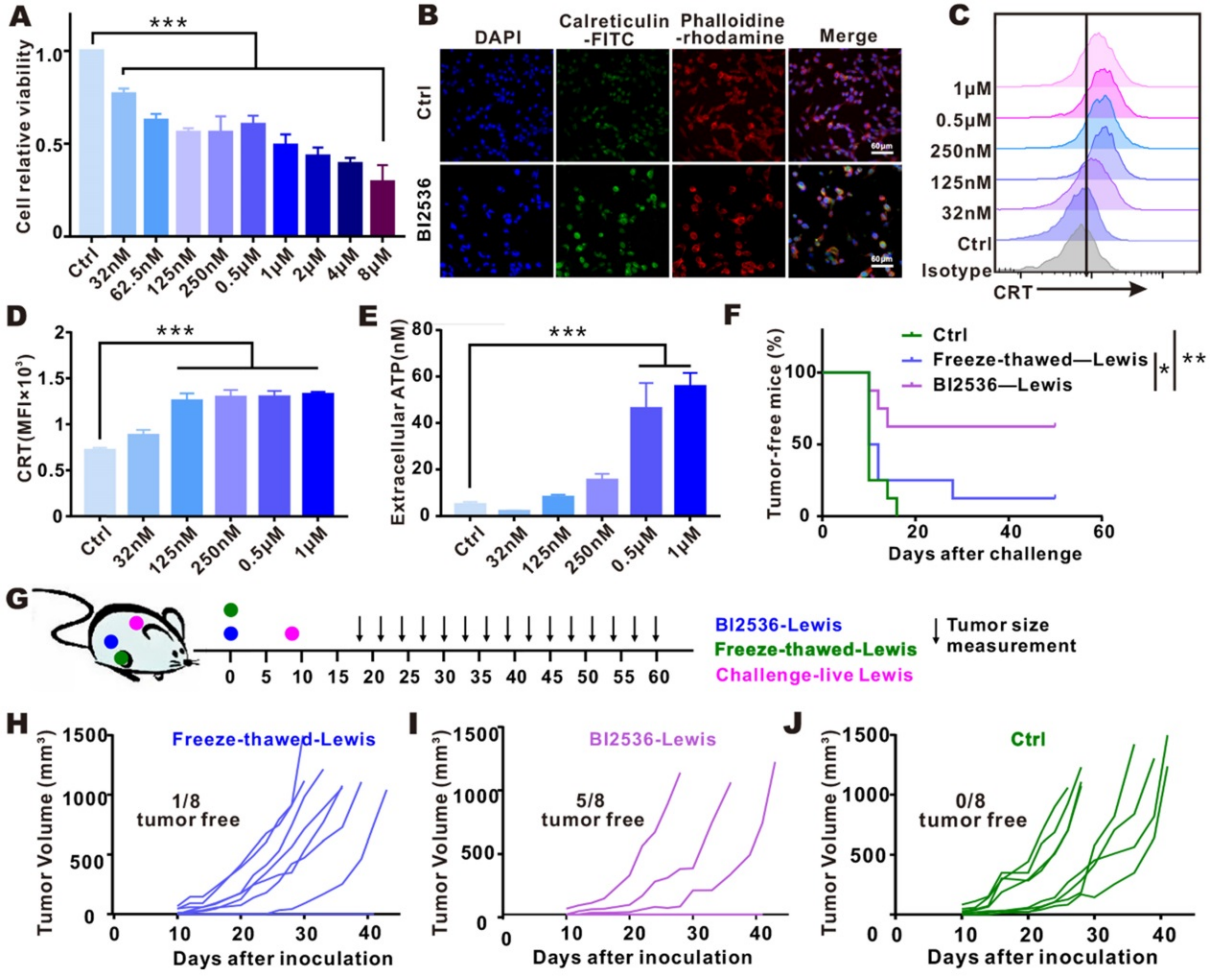
** Targeting PLK1 elicited ICD *in vitro* and *in vivo*. A-E.** LLC cells were treated for 24 h with indicated concentrations of BI2536. A. Cell viability was measured by CCK-8 assay. **B.** Immunofluorescence imaging of CRT translocation (green), phalloidin (red, cellular cytoskeleton) and DAPI (blue, nuclei). Scale bars, 60 µm. **C-D.** The data of CRT expression by Flow cytometry. **E.** Release of ATP in cell culture supernatants. **F-J.** LLC cells treated *in vitro* with BI2536 or by freezing-thawing were inoculated into right flank of immunocompetent C57BL/6 mice. Eight days after inoculation, the mice were challenged with live LLC cells in the opposite flank, as indicated in treatment scheme (G). The survival condition and tumor size were monitored every 2-3 days. **F.** Percentage of tumor-free mice in each group (*n*=8). **H-J.** Growth of challenged tumors in an established immunization model.

Since PLK1 inhibition was shown to promote APC activation *in vivo*, and bioinformatic analysis exhibited that antigen processing and presentation pathway was enriched in tumor tissues with low PLK1 expression, we were led to hypothesize that the activation of antitumor immunity by PLK1 blockade might be achieved through its modulation on DC function. To test this hypothesis, we examined the expression of costimulatory molecules on the surface of BMDCs and their phagocytic function. As shown in Fig. S3, the direct treatment of BMDCs with BI2536 could hardly enhance their activation and engulfment, indicating that an indirect regulation might serve as a link between PLK1 blockade and DCs. Usually, phagocytosis of tumor cells that have undergone ICD may promote DC activation and effector T cell priming, and multiple anti-neoplastic agents have been confirmed to induce ICD 25, 26. To see if ICD takes place in tumor cells after treatment with BI2536, we examined the release or cell-surface expression of danger-related molecular patterns (DAMPs) by the dying tumor cells *in vitro*. As shown in Fig. 3A, cell proliferation was inhibited by BI2536 in a dose-dependent manner, which was in line with the elevated expression of tumor cell-surface CRT and increased secretion of ATP (Fig. 3B-E).

Furthermore, to confirm that the agent is capable of inducing ICD, vaccination assay, the gold-standard approach, was also conducted 21. To prepare a putative DC vaccine, we subcutaneously inoculated BI2536-killed tumor cells into the flank of immunocompetent-C57BL/6 mice. For setting up controls, tumor cells subjected to freezing-thawing were injected in the same way. 8 days after the inoculation, living cancer cells of the same type were challenged in the contralateral flank, as indicated in Fig. 3G. Compared with other groups, percentage of tumor-free mice was obviously higher after vaccination with BI2536-killed tumor cells (Fig. 3F), which was in line with less dramatic increase in tumor size (Fig. 3H-J), suggesting that BI2536 worked more efficiently in triggering *bona fide* ICD.

### PLK1 blocker-treated tumor cells enhanced phagocytosis and promoted maturation of BMDCs

**Figure 4:**
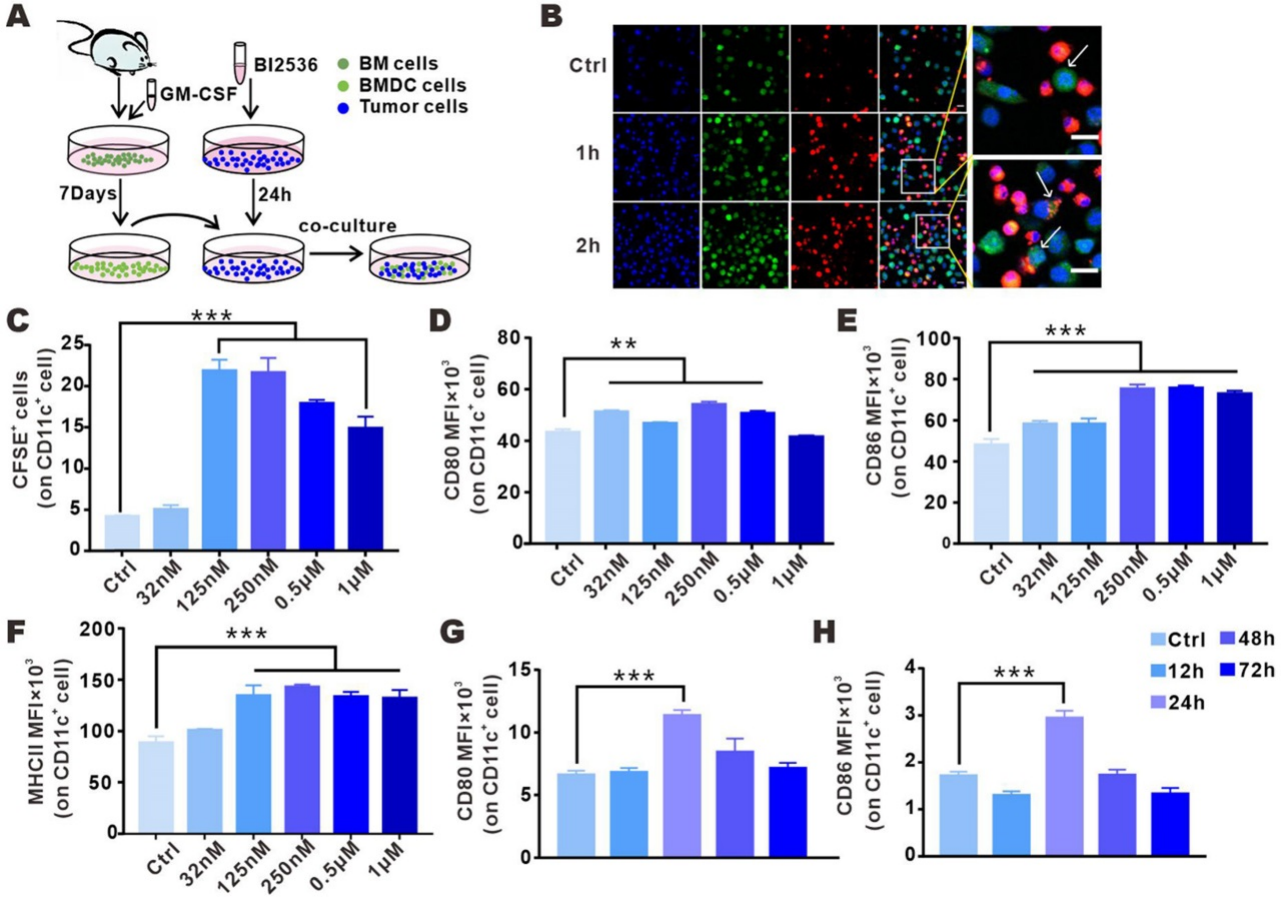
** BI2536-treated tumor cells were phagocytosed and could promote BMDC activation. A.** Schematic diagram of co-culture system illustrating the effect of BI2536-treated tumor cells on BMDC function. **B.** Representative immunofluorescence images showing nucleus (blue, DAPI), BMDCs (red, DiI) and BI2536-treated LLC cells (green, CFSE) 0, 1 and 2 h after co-culture (confocal imaging, objective: 20×). The arrow indicates the surrounding BMDCs ready to engulf tumor cells. Scale bars, 20 µm. **C.** The phagocytosis of CD11c^+^ DCs after co-culture with BI2536-treated LLC cells (CFSE), as assessed by flow cytometry. **D-F.** The expression of CD80, CD86 and MHCII in BMDC after incubation with treated tumor cells. **G-H.** LLC cells treated with BI2536 were inoculated subcutaneously into mice and the activation of DCs in inguinal lymph nodes was flow cytometrically detected at different time points after vaccination. The MFI of CD80 and CD86 on CD11c^+^ cells are listed.

ICD renders tumor antigens “visible” to immune system since CRT carries the “eat me” signal that is translocated from the lumen of endoplasmic reticulum to tumor surface, which substantially facilitates the phagocytosis of tumor cells by DCs 20. To assess the susceptibility of dying cells to phagocytosis by DCs, we incubated CFSE-labeled tumor cells in DMEM with or without BI2536, and then co-cultured the treated tumor cells with BMDCs (Fig. 4A). Confocal analysis showed that a strong interaction existed between BMDCs and drug-treated tumor cells. As shown in Fig. 4B, after only 1 h of co-culture, BMDCs began to surround the tumor cells, and the phagocytic signal was detected 2 h after the co-culture. In order to more accurately determine the level of the engulfing, phagocytosis was flow cytometrically measured, with the phagocytosed tumor cells defined as the cells positive for both CD11c and CFSE. 24 h after co-incubation, the phagocytosis of BMDCs on BI2536-treated tumor cells was found to be 4-fold higher as compared with the control group (Fig. 4C). Given that cells having undergone ICD could promote DC maturation 26-28, we assessed the effect of PLK1 blockade on this process in the aforementioned co-culture system. As shown in Fig. 4D-F, surface expression of CD80, CD86 and MHCII was increased in BMDCs after co-cultured with BI2536-treated cells, suggesting that BI2536 was more efficient than its isotype control in inducing DC maturation.

In tumor models, localized tumor antigens were mostly phagocytosed by DCs and the antigen-loaded DCs migrated to draining LNs for a full activation and then, triggered antitumor adaptive immune response 15, 29. To determine whether BI2536-related ICD can promote DC maturation in tumor-draining LNs, dying tumor cells treated by BI2536 were injected subcutaneously into the tail rote of immunocompetent normal mice. 12-72 h after the injection, the expression of costimulatory ligands in DCs from draining inguinal lymph nodes were analyzed by flow cytometry. Consistent with the above-mentioned *in vitro* results, tumor cells pretreated with BI2536 were more likely to elevate the level of DC activation markers in comparison with frozen-thawed tumor cells (Fig. 4G-H). Collectively, these results suggested that BI2536 treatment could increase DC maturation, which enhances antigen processing and presentation in antitumor T cell priming.

## Discussion

Since PLK1 can drive cell cycle progression, blocking PLK1 has been extensively explored as an antitumor strategy for various tumor types, including NSCLC 30-33. PLK1 inhibitors are generally believed to be able to induce tumor apoptosis after arresting cell cycle 10. However, whether and how the inhibitors work via immune modulation remain unknown.

In the present study, we demonstrated that blocking PLK1 could modulate the immune microenvironment within tumors by inducing ICD, thereby promoting T cells-based antitumor immune priming. First, we found that PLK1 was correlated negatively with multiple immune cell lineages and played a crucial role in antigen processing and presentation in LUAD. Second, we found that inhibiting PLK1 promoted immune cell infiltration and activation *in vivo*. Third, PLK1 blockade was found to trigger DC activation in an indirect fashion by inducing tumor cell death in an immunogenic manner, and vaccination with BI2536-killed tumor cells helped suppress tumor growth. Last, we revealed that the improved immune response was a result of increased antigen presentation of DCs, characterized by enhanced phagocytosis and activation of DCs.

DC activation is generally believed to be an essential event for antigen processing and for provision of costimulatory signals for activation of T cells 15. We found that PLK1 inhibition promoted activation and function of T cells and DC cells *in vivo.* Further bioinformatic analysis suggested that tumor tissues over-expressing PLK1 downregulated the antigen processing and presentation pathways. In view of these results, we hypothesized that activated immunity caused by PLK1 blockade might be, ascribed to direct or indirect regulation of DC function. Given that DC activation caused by microtubular destabilization increases the infiltration of effector T cells within tumors 34-36, it is likely that PLK1, as a modulator of microtubular dynamics, might be implicated in DC maturation. In this study, we detected the phagocytosis and maturation of BMDCs after treatment with BI2536. As shown in Fig S3, BI2536 could barely enhance the activation or engulfment of BMDCs, suggesting that BI2536 might modulate DCs in an indirect rather than a direct manner.

Depending on the differential signals generated by dying cells, cancer cell death can be of either non-immunogenic or immunogenic nature 37. ICD, a “visible” immunogenic death, hallmarked by release of DAMPs, is seen as a powerful DC activator. In our study, we found that, after treatment with BI2536, the increased release of DAMPs enhanced the phagocytosis of DCs and subsequent presentation of tumor-specific antigens. Nonetheless, DAMPs are not the sole indicator for determining ICD, since cardiac glycosides alone fail to elicit immunological memory responses even if they have promoted CRT exposure and ATP secretion in tumor cells 38. For definitive identification of a *bona fide* ICD inducer, vaccination assay is deemed as the gold-standard approach 21. In agreement with this finding, we found the enhanced immune memory responses in challenged model of vaccinated (BI2536-treated tumor cell) mice. Our results suggested that PLK1 blockade indirectly elicited the activation of DCs to prime T cells, possibly via ICD-related increase in phagocytosis of specific tumor antigens and expression of costimulatory molecules on DCs.

PLK1 might be also involved in the immunosuppressive mechanisms by modulating checkpoint inhibition. As shown in Fig 1F, PLK1 was correlated positively with the expression of multiple immunosuppressive checkpoints, suggesting that the inhibitory signals on immune response might be weakened after PLK1 is targeted. PLK1 blockade reportedly could lower the expression of Myc in tumor cells, since Myc inactivation has been demonstrated to induce the down-regulation of PDL1, an immunosuppressive molecule 39-42, which was identified as the major trigger of T cell exhaustion. In addition, PLK1 might be an attractive target to activate DCs by regulating immune checkpoints, thereby further inducing T-cell responses. Involved in tryptophan metabolism, IDO1 could promote the formation of Tregs by generating immune-tolerant DCs 43-44. Moreover, LAG3 could exhaust T cells by modulating the intensity of the antigenic stimuli in DCs 45. Therefore, the elevated anti-tumor immunological effect might be partially attributed to the relief of immunosuppressive effects after targeting PLK1. Nevertheless, PLK1 blockade cannot induce persisting regression of tumor, since the drug-treated vaccine stimulates a transient not a lasting DC activation (Fig. 4H) unless the vaccine is repeatedly administered. Considering that the immunogenic chemotherapy alone did not suffice to trigger antitumor immunity in immunosuppressive tumor microenvironment (TME) 46, it is possible that the promoting effect of BI2536 on immune activation was partially compromised by the network of other immunosuppressive components, such as myeloid-derived suppressor cells (MDSCs), tumor-associated macrophages (TAMs), neutrophils, among others. Moreover, another contributor might be the limited immune response which could not eradicate tumors altogether.

In conclusion, this study identified a novel role of PLK1 blockade, *i.e*., an immune activator. We also showed that PLK1 blockade elicited an indirect DC activation and T cell priming by inducing ICD. Nevertheless, whether some other mechanisms are implicated in the activation of immunity was not explored in-depth in the study, nor was the specific signaling pathway in ICD inducing was examined. To overcome the limitations, in future, we will combine *in vivo* imaging, single-cell sequencing, spatial transcriptome and other technologies to clarify the specific mechanism. Moreover, the strategies that integrate PLK1 blockade and tumor-killers or immunomodulators, which promote the antitumor cytotoxicity and awaken immunity against immunosuppressive TME, might be a promising approach for maximizing the effect of PLK1 blockade in future.

## Supplementary Material

Supplementary figures and tables.

Supplementary tables.

## Abbreviations

NSCLC: non-small cell lung cancer
ICD: immunogenic cell death
PLK: polo-like kinase
AURKA: aurora kinase A
CRT: calreticulin
LUAD: lung adenocarcinoma
LLC: Lewis
PFA: paraformaldehyde
BMDCs: Mouse bone marrow-derived DCs
DAMPs: danger-related molecular patterns
ILNs: inguinal lymph glands
APC: antigen presenting cell
TME: tumor microenvironment
MDSCs: myeloid-derived suppressor cells
TAMs: tumor-associated macrophages

## References

[1] Strebhardt K, Ullrich A (2006). Targeting polo-like kinase 1 for cancer therapy. Nat Rev Cancer.

[2] Eckerdt F, Yuan J, Strebhardt K (2005). Polo-like kinases and oncogenesis. Oncogene.

[3] Gheghiani L, Wang L, Zhang Y, Moore XTR, Zhang J, Smith SC (2020). PLK1 induces chromosomal instability and overrides cell cycle checkpoints to drive tumorigenesis. Cancer Res.

[4] Wolf G, Elez R, Doermer A, Holtrich U, Ackermann H, Stutte HJ (1997). Prognostic significance of polo-like kinase (PLK) expression in non-small cell lung cancer. Oncogene.

[5] Jeong SB, Im JH, Yoon JH, Bui QT, Lim SC, Song JM (2018). Essential Role of Polo-like Kinase 1 (Plk1) Oncogene in Tumor Growth and Metastasis of Tamoxifen-Resistant Breast Cancer. Mol Cancer Ther.

[6] Fu Z, Wen D (2017). The Emerging Role of Polo-Like Kinase 1 in Epithelial-Mesenchymal Transition and Tumor Metastasis. Cancers (Basel).

[7] Lens SM, Voest EE, Medema RH (2010). Shared and separate functions of polo-like kinases and aurora kinases in cancer. Nat Rev Cancer.

[8] Yin T, Zhao ZB, Guo J, Wang T, Yang JB, Wang C (2019). Aurora A Inhibition Eliminates Myeloid Cell-Mediated Immunosuppression and Enhances the Efficacy of Anti-PD-L1 Therapy in Breast Cancer. Cancer Res.

[9] Lénárt P, Petronczki M, Steegmaier M, Di Fiore B, Lipp JJ, Hoffmann M (2007). The small-molecule inhibitor BI 2536 reveals novel insights into mitotic roles of polo-like kinase 1. Curr Biol.

[10] Shin SB, Woo SU, Yim H (2015). Differential Cellular Effects of Plk1 Inhibitors Targeting the ATP-binding Domain or Polo-box Domain. J Cell Physiol.

[11] Steegmaier M, Hoffmann M, Baum A, Lénárt P, Petronczki M, Krssák M (2007). BI 2536, a potent and selective inhibitor of polo-like kinase 1, inhibits tumor growth *in vivo*. Curr Biol.

[12] Wu M, Wang Y, Yang D, Gong Y, Rao F, Liu R (2019). A PLK1 kinase inhibitor enhances the chemosensitivity of cisplatin by inducing pyroptosis in oesophageal squamous cell carcinoma. EBioMedicine.

[13] Wang Q, Wang Y, Ding J, Wang C, Zhou X, Gao W (2020). A bioorthogonal system reveals antitumour immune function of pyroptosis. Nature.

[14] Li M, Liu Z, Wang X (2018). Exploration of the Combination of PLK1 Inhibition with Immunotherapy in Cancer Treatment. J Oncol.

[15] Gardner A, Ruffell B (2016). Dendritic Cells and Cancer Immunity. Trends Immunol.

[16] Bezu L, Gomes-de-Silva LC, Dewitte H, Breckpot K, Fucikova J, Spisek R (2015). Combinatorial strategies for the induction of immunogenic cell death. Front Immunol.

[17] Fucikova J, Kralikova P, Fialova A, Brtnicky T, Rob L, Bartunkova J (2011). Human tumor cells killed by anthracyclines induce a tumor-specific immune response. Cancer Res.

[18] Rodriguez-Ruiz ME, Vitale I, Harrington KJ, Melero I, Galluzzi L (2020). Immunological impact of cell death signaling driven by radiation on the tumor microenvironment. Nat Immunol.

[19] Krombach J, Hennel R, Brix N, Orth M, Schoetz U, Ernst A (2019). Priming anti-tumor immunity by radiotherapy: Dying tumor cell-derived DAMPs trigger endothelial cell activation and recruitment of myeloid cells. Oncoimmunology.

[20] Zitvogel L, Kepp O, Senovilla L, Menger L, Chaput N, Kroemer G (2010). Immunogenic tumor cell death for optimal anticancer therapy: the calreticulin exposure pathway. Clin Cancer Res.

[21] Galluzzi L, Buqué A, Kepp O, Zitvogel L, Kroemer G (2017). Immunogenic cell death in cancer and infectious disease. Nat Rev Immunol.

[22] Huang DW, Sherman BT, Lempicki RA (2009). Systematic and integrative analysis of large gene lists using DAVID bioinformatics resources. Nat Protoc.

[23] Becht E, Giraldo NA, Lacroix L, Buttard B, Elarouci N, Petitprez F (2016). Estimating the population abundance of tissue-infiltrating immune and stromal cell populations using gene expression. Genome Biol.

[24] Ribas A, Wolchok JD (2018). Cancer immunotherapy using checkpoint blockade. Science.

[25] Liu P, Zhao L, Pol J, Levesque S, Petrazzuolo A, Pfirschke C (2019). Crizotinib-induced immunogenic cell death in non-small cell lung cancer. Nat Commun.

[26] Hossain DMS, Javaid S, Cai M, Zhang C, Sawant A, Hinton M (2018). Dinaciclib induces immunogenic cell death and enhances anti-PD1-mediated tumor suppression. J Clin Invest.

[27] Pozzi C, Cuomo A, Spadoni I, Magni E, Silvola A, Conte A (2016). The EGFR-specific antibody cetuximab combined with chemotherapy triggers immunogenic cell death. Nat Med.

[28] Nam GH, Lee EJ, Kim YK, Hong Y, Choi Y, Ryu MJ (2018). Combined Rho-kinase inhibition and immunogenic cell death triggers and propagates immunity against cancer. Nat Commun.

[29] Chen DS, Mellman I (2013). Oncology meets immunology: the cancer-immunity cycle. Immunity.

[30] Sebastian M, Reck M, Waller CF, Kortsik C, Frickhofen N, Schuler M (2010). The efficacy and safety of BI 2536, a novel Plk-1 inhibitor, in patients with stage IIIB/IV non-small cell lung cancer who had relapsed after, or failed, chemotherapy: results from an open-label, randomized phase II clinical trial. J Thorac Oncol.

[31] Dill V, Kauschinger J, Hauch RT, Buschhorn L, Odinius TO, Müller-Thomas C (2020). Inhibition of PLK1 by capped-dose volasertib exerts substantial efficacy in MDS and sAML while sparing healthy haematopoiesis. Eur J Haematol.

[32] El Dika I, Lim HY, Yong WP, Lin CC, Yoon JH, Modiano M (2019). An Open-Label, Multicenter, Phase I, Dose Escalation Study with Phase II Expansion Cohort to Determine the Safety, Pharmacokinetics, and Preliminary Antitumor Activity of Intravenous TKM-080301 in Subjects with Advanced Hepatocellular Carcinoma. Oncologist.

[33] Weiss GJ, Jameson G, Von Hoff DD, Valsasina B, Davite C, Di Giulio C (2018). Phase I dose escalation study of NMS-1286937, an orally available Polo-Like Kinase 1 inhibitor, in patients with advanced or metastatic solid tumors. Invest New Drugs.

[34] Müller P, Kreuzaler M, Khan T (2015). Trastuzumab emtansine (T-DM1) renders HER2+ breast cancer highly susceptible to CTLA-4/PD-1 blockade. Sci Transl Med.

[35] Martin K, Müller P, Schreiner J (2014). The microtubule-depolymerizing agent ansamitocin P3 programs dendritic cells toward enhanced anti-tumor immunity. Cancer Immunol Immunother.

[36] Müller P, Martin K, Theurich S (2014). Microtubule-depolymerizing agents used in antibody-drug conjugates induce antitumor immunity by stimulation of dendritic cells. Cancer Immunol Res.

[37] Green DR, Ferguson T, Zitvogel L, Kroemer G (2009). Immunogenic and tolerogenic cell death. Nat Rev Immunol.

[38] Menger L, Vacchelli E, Adjemian S, Martins I, Ma Y, Shen S (2012). Cardiac glycosides exert anticancer effects by inducing immunogenic cell death. Sci Transl Med.

[39] Xiao D, Yue M, Su H, Ren P, Jiang J, Li F (2016). Polo-like Kinase-1 Regulates Myc Stabilization and Activates a Feedforward Circuit Promoting Tumor Cell Survival. Mol Cell.

[40] Ren Y, Bi C, Zhao X, Lwin T, Wang C, Yuan J (2018). PLK1 stabilizes a MYC-dependent kinase network in aggressive B cell lymphomas. J Clin Invest.

[41] Higuchi F, Fink AL, Kiyokawa J, Miller JJ, Koerner MVA, Cahill DP (2018). PLK1 Inhibition Targets Myc-Activated Malignant Glioma Cells Irrespective of Mismatch Repair Deficiency-Mediated Acquired Resistance to Temozolomide. Mol Cancer Ther.

[42] Casey SC, Tong L, Li Y, Do R, Walz S, Fitzgerald KN (2016). MYC regulates the antitumor immune response through CD47 and PD-L1. Science.

[43] Cheong JE, Sun L (2018). Targeting the IDO1/TDO2-KYN-AhR Pathway for Cancer Immunotherapy-Challenges and Opportunities. Trends Pharmacol Sci.

[44] Munn DH, Mellor AL (2016). IDO in the Tumor Microenvironment: Inflammation, Counter-Regulation, and Tolerance. Trends in immunology.

[45] Lichtenegger FS, Rothe M, Schnorfeil FM (2018). Targeting LAG-3 and PD-1 to Enhance T Cell Activation by Antigen-Presenting Cells. Frontiers in immunology.

[46] Ciampricotti M, Hau CS (2012). Chemotherapy response of spontaneous mammary tumors is independent of the adaptive immune system. Nat Med.

