# Supplementary material for: PLK1 Inhibition Induces Immunogenic Cell Death and Enhances Immunity against NSCLC
Source: Int J Med Sci. 2021 Aug 19;18(15):3516–25. doi: 10.7150/ijms.60135 (PMC8436107; doi:10.7150/ijms.60135)
Supplement: Supplementary file 1 — Supplementary figures and tables. [file ijmsv18p3516s1.pdf]

PLK1 Inhibition Induces Immunogenic Cell Death and Enhances Immunity Against NSCLC

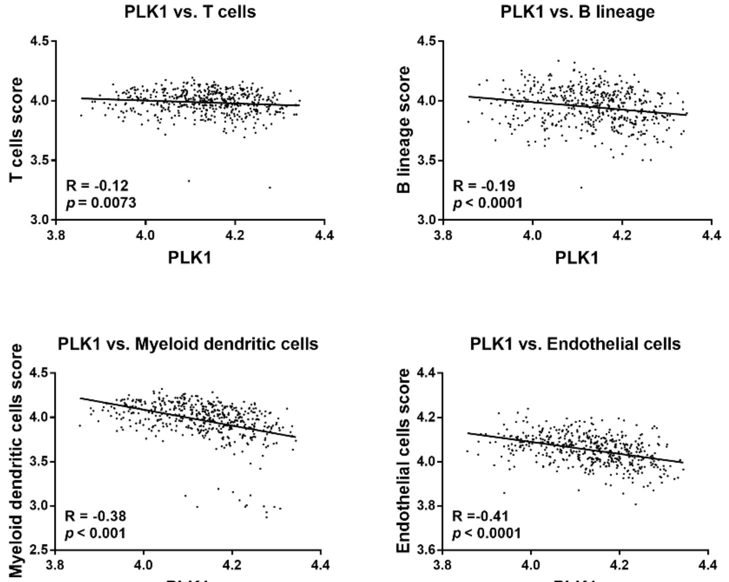

Fig S1. Negative correlation between PLK1 expression and infiltrating levels of T cells, B lineage, myeloid dendritic cells and endothelial cells in LUAD mined from the TCGA database.

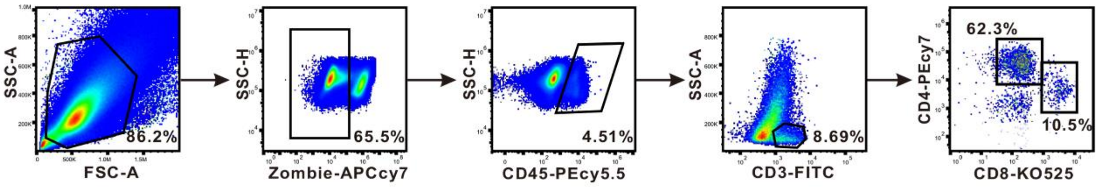

Fig S2. Gating strategies for the detection ratio of CD4^{+}/CD8^{+} T cells.

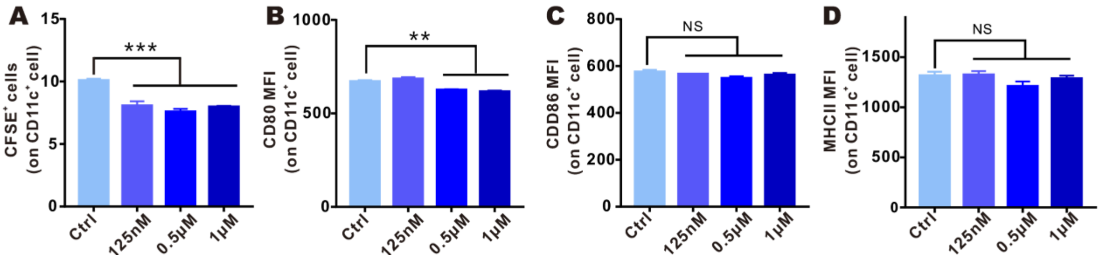

Fig S3. BI2536 could hardly enhance DC function.

A. The percentage of CD11c^{+} BMDCs with engulfed Lewis cells after treatment with

BI2536.

B-D. Surface expression of CD80, CD86 and MHCII on BI2536-treated $CD11c^+$ DCs.

Supplementary Table 1. Multiple Comparisons of the PLK1 expressions at stages 1-4

LSD

(I) Stage	(J) Stage	Mean difference (I-J)	Std. Error	Sig.	95% CI	
					Lower Bound	Upper Bound
1	2	-.03918*	0.0107	0	-0.0602	-0.0181
	3	-.04520*	0.01236	0	-0.0695	-0.0209
	4	-.06015*	0.01996	0.003	-0.0994	-0.0209
2	1	.03918*	0.0107	0	0.0181	0.0602
	3	-0.00602	0.01409	0.669	-0.0337	0.0217
	4	-0.02097	0.02107	0.32	-0.0624	0.0204
3	1	.04520*	0.01236	0	0.0209	0.0695
	2	0.00602	0.01409	0.669	-0.0217	0.0337
	4	-0.01495	0.02196	0.496	-0.0581	0.0282
4	1	.06015*	0.01996	0.003	0.0209	0.0994
	2	0.02097	0.02107	0.32	-0.0204	0.0624
	3	0.01495	0.02196	0.496	-0.0282	0.0581

*. The mean difference is significant at the 0.05 level.

LSD= least significance difference

Supplementary Table 2. ANOVA analysis of the PLK1 expression at stages 1-4

	Sum of squares	df	Mean square	F	Sig.
Between Groups	0.246	3	0.082	8.67	0
Within Groups	4.683	495	0.009		
Total	4.929	498			

Supplementary Table 3. Multiple Comparisons of the PLK1 expression at stages 2-4

LSD

(I)Stage	(J) Stage	Mean difference (I-J)	Std. Error	Sig.	95% CI	
					Lower Bound	Upper Bound
2	3	-0.00602	0.01352	0.657	-0.0327	0.0206
	4	-0.02097	0.02022	0.301	-0.0608	0.0189
3	2	0.00602	0.01352	0.657	-0.0206	0.0327
	4	-0.01495	0.02107	0.479	-0.0565	0.0266
4	2	0.02097	0.02022	0.301	-0.0189	0.0608
	3	0.01495	0.02107	0.479	-0.0266	0.0565

LSD= least significance difference

Supplementary Table 4. ANOVA analysis of the PLK1 expression at stages 2-4

	Sum of squares	df	Mean square	F	Sig.
Between Groups	0.01	2	0.005	0.553	0.576
Within Groups	1.925	221	0.009		
Total	1.935	223			

Supplementary Table 5. Correlations between PLK1 and immune checkpoints

Variable 1	Variable 2	Pearson correlation coefficient	P value
PLK1	PD1	0.1561	0.0005
PLK1	PDL1	0.2644	<0.0001
PLK1	LAG3	0.1779	<0.0001
PLK1	SIGLEC10	0.1673	0.0002
PLK1	IDO1	0.1682	0.0002
PD1	PDL1	0.4063	<0.0001
PD1	LAG3	0.613	<0.0001
PD1	SIGLEC10	0.554	<0.0001
PD1	IDO1	0.238	<0.0001
PDL1	LAG3	0.2966	<0.0001
PDL1	SIGLEC10	0.4036	<0.0001
PDL1	IDO1	0.302	<0.0001
LAG3	SIGLEC10	0.4968	<0.0001
LAG3	IDO1	0.3463	<0.0001
SIGLEC10	IDO1	0.2761	<0.0001
